# Exposure to Arsenic and Subclinical Cardiovascular Disease in 9- to 11-Year-Old Children, Syracuse, New York

**DOI:** 10.1001/jamanetworkopen.2023.21379

**Published:** 2023-06-30

**Authors:** Brooks B. Gump, Kevin Heffernan, Lynn S. Brann, Dustin T. Hill, Charlotte Labrie-Cleary, Vikrant Jandev, James A. MacKenzie, Nader H. Atallah-Yunes, Patrick J. Parsons, Christopher D. Palmer, Austin A. Roberts, Kestutis Bendinskas

**Affiliations:** 1 Department of Public Health, Syracuse University, Syracuse, New York; 2 Department of Exercise Science, Syracuse University, Syracuse, New York; 3 Department of Nutrition and Food Studies, Syracuse University, Syracuse, New York; 4 Department of Chemistry, State University of New York College at Oswego, Oswego; 5 Department of Biological Sciences, State University of New York College at Oswego, Oswego; 6 Department of Pediatrics, SUNY Upstate Medical University, Syracuse, New York; 7 Laboratory of Inorganic and Nuclear Chemistry, Wadsworth Center, New York State Department of Health, Albany; 8 Department of Environmental Health Sciences, School of Public Health, University at Albany, Albany, New York

## Abstract

**Importance:**

Studies in adults have demonstrated associations between arsenic exposure and clinical and subclinical cardiovascular disease (CVD). No studies to date have considered potential associations in children.

**Objective:**

To examine the association between total urinary arsenic levels in children and subclinical indicators of CVD.

**Design, Setting, and Participants:**

This cross-sectional study considered 245 children, a subset from the Environmental Exposures and Child Health Outcomes (EECHO) cohort. Children from the Syracuse, New York, metropolitan area were recruited from August 1, 2013, until November 30, 2017, with enrollment throughout the year. Statistical analysis was performed from January 1, 2022, to February 28, 2023.

**Exposures:**

Total urinary arsenic was measured using inductively coupled plasma mass spectrometry. Creatinine concentration was used to adjust for urinary dilution. In addition, potential exposure routes (eg, diet) were measured.

**Main Outcomes and Measures:**

Three indicators of subclinical CVD were assessed: carotid-femoral pulse wave velocity, carotid intima media thickness, and echocardiographic measures of cardiac remodeling.

**Results:**

The study sample included 245 children aged 9 to 11 years (mean [SD] age, 10.52 [0.93] years; 133 [54.3%] female). The geometric mean of the creatinine-adjusted total arsenic level in the population was 7.76 μg/g creatinine. After adjustment for covariates, elevated total arsenic levels were associated with significantly greater carotid intima media thickness (β = 0.21; 95% CI, 0.08-0.33; *P* = .001). In addition, echocardiography revealed that elevated total arsenic was significantly higher for children with concentric hypertrophy (indicated by greater left ventricular mass and greater relative wall thickness; geometric mean, 16.77 μg/g creatinine; 95% CI, 9.87-28.79 μg/g) relative to the reference group (geometric mean, 7.39 μg/g creatinine; 95% CI, 6.36-8.58 μg/g). With respect to exposure source, significant geographic clustering of total arsenic was found in 1 urban area of Syracuse, New York.

**Conclusions and Relevance:**

These findings suggest a significant association between arsenic exposure and subclinical CVD in children. Elevated total arsenic levels were found in an area of Syracuse with known elevations of toxic metals from industrial waste, suggesting historical pollution as a possible source. Given the novelty and potential importance of this association, further research is needed to confirm our findings. Any potential effect of urinary arsenic exposure in childhood on actual clinical CVD outcomes in adulthood remains to be determined.

## Key Points

**Question:**

What is the association between total arsenic in urine and subclinical cardiovascular disease in children?

**Findings:**

In this cross-sectional study of 245 children aged 9 to 11 years, higher total arsenic was geographically clustered and associated with significantly greater carotid intima media thickness as well as concentric cardiac hypertrophy.

**Meaning:**

These findings suggest that arsenic exposure may increase the risk of subclinical cardiovascular disease in children residing in certain areas with historic pollution in Syracuse.

## Teaser

This cross-sectional study of children in Syracuse, New York, investigates the association between arsenic exposure and subclinical cardiovascular disease.

## Introduction

Numerous studies^1,2,3^ have reported associations between arsenic exposure in adults and cardiovascular disease (CVD), including coronary heart disease, stroke, and peripheral arterial disease. In addition, arsenic exposure is linked to subclinical CVD indexes, such as carotid intima media thickness (cIMT)^4^, left ventricular mass (LVM), and hypertrophy.^5^ Mechanisms that might explain these arsenic-CVD associations include inflammation in vascular tissues, oxidative stress, endothelial injury, and smooth-muscle cell proliferation.^6^

Although the association between arsenic and CVD is relatively well established in adults, research in children is limited. Children are an important population to consider because children can be more sensitive to the effects of toxicants,^7^ and CVD risks measured in childhood are associated with cardiovascular events and deaths in adulthood.^8^ In addition, identifying subclinical risks at a young age enables us to consider earlier interventions and thereby exert greater influence on long-term CVD risk.^9^ Two studies^10,11^ have shown associations between external arsenic exposure assessments in children (eg, levels in drinking water and years residing near smelting) and CVD risks. We are unaware of any studies in children assessing the body burden of arsenic (eg, total urinary arsenic) in association with clinical or subclinical CVD.

The current study considered the association between total arsenic and subclinical CVD within the Environmental Exposures and Child Health Outcomes (EECHO) cohort.^12,13,14^ Within this cohort, we measured total arsenic, subclinical CVD (intima media thickness [IMT], pulse wave velocity [PWV], and echocardiography-determined cardiac remodeling), and important potential confounders, such as race and socioeconomic status (SES). Racial variation in arterial stiffness is an established finding seen across the life span, hence our inclusion of race as a covariate. Socioeconomic status is an established risk factor for CVD; therefore, we also considered SES as a potential covariate. Arsenic is considered cardiotoxic,^15^ and, based on prior research with adults,^3^ we hypothesized that increasing total arsenic in children would be significantly associated with increasing subclinical CVD as indexed by cIMT and carotid-femoral pulse wave velocity (cfPWV). We found no literature (in children or adults) that has addressed arsenic exposure in relation to specific patterns of potential cardiac remodeling.^16^ Given the arsenic-LVM association found in adults^5^ but absent prior research on arsenic and relative wall thickness (RWT), we hypothesized that arsenic exposure in children would be associated with either concentric hypertrophy (elevated LVM and RWT) or eccentric hypertrophy (elevated LVM but normal RWT). Concentric hypertrophy is a particularly important outcome measure because it is strongly associated with future cardiac events.^17^

As a secondary aim, we evaluated potential exposure routes, including diet, drinking water, geographic location, and secondhand smoke. To help meet this aim, we considered the relative contribution of dietary organoarsenic species (generally considered benign and ingested through diet) and inorganic arsenic species (generally elevated as a function of geographic location and industrial contamination) in a subsample. Total arsenic in urine in the US population is primarily composed of dietary organoarsenic species (primarily arsenobetaine), methylated inorganic arsenic species (primarily dimethylated arsenic acid), and unmetabolized inorganic species of arsenic (arsenate and arsenite).^18^

## Methods

### Participants

The cross-sectional EECHO study included an approximately equal number of Black and White and male and female children. Children were excluded who did not self-identify as either Black or White (the purpose of the EECHO study was to explore relationships between environmental toxicants and CVD risk in a biracial cohort of children in Syracuse, New York, because the demographic composition of Syracuse is approximately 47% White and 30% African American/Black), were not 9 to 11 years old, and did not meet zip code residence criteria (designed to target low-SES neighborhoods having roughly equal numbers of Black and White children). Recruitment occurred from August 1, 2013, until November 30, 2017, with enrollment throughout the year. Only participants providing urine were included; however, there were no significant differences in age, race, sex, height, SES, and body mass index (BMI; calculated as weight in kilograms divided by height in meters squared) between those included with valid total arsenic (N = 245) and those not included (n = 54). Statistical analyses were further restricted to those with valid outcomes for cIMT (n = 227), cfPWV (n = 214), or LVM and RWT (n = 244). Reasons for data loss were scheduling difficulties, staff shortages, and/or technical issues. The parent or caregiver informant was usually the mother (86%) but occasionally a father (9%), grandmother (2%), or another custodian (eg, aunt) (3%). Participants arrived at our laboratory and signed an assent form while a parent signed a separate consent form approved by the Syracuse University Institutional Review Board. This study followed the Strengthening the Reporting of Observational Studies in Epidemiology (STROBE) reporting guideline.

### Procedure

Participants’ spot urine was collected as during any typical physician visit (ie, collection cup left in bathroom for child). We scheduled separate visits to a vascular laboratory (to assess cIMT and cfPWV) and a pediatric cardiologist (N.H.A.-Y.) for echocardiography to assess LVM and RWT. Surveys were administered using iPads and Qualtrics Survey software, February 2013 to November 2016 (Qualtrics XM). Additional information regarding our study procedures can be found in eAppendix 1 in Supplement 1.

### Measures

#### Arsenic in Urine

Urine samples were stored at −80 °C and shipped on dry ice to the Laboratory of Inorganic and Nuclear Chemistry at the Wadsworth Center, New York State Department of Health for analysis of total arsenic using inductively coupled plasma mass spectrometry calibrated with National Institute of Standards and Technology–traceable standards (PerkinElmer).^19^ We randomly selected 32 urine samples (13.1% of the cohort) from those with total arsenic levels exceeding 20 μg/g and conducted arsenic speciation analysis. Five arsenic species were quantified in urine: arsenocholine, arsenobetaine, monomethlyarsonic acid, dimethylated arsenic acid, and unmetabolized inorganic arsenic species. For purposes of analysis in this study, we summed arsenobetaine and arsenocholine for quantification of dietary organic arsenic and monomethlyarsonic acid, dimethylated arsenic acid, and inorganic arsenic species for quantification of total inorganic (metabolized and unmetabolized) arsenic.

#### Urinary Creatinine

To assess urinary creatinine concentrations, samples were diluted 20-fold and measured in duplicate using a creatinine colorimetric detection kit (Enzo Life Sciences) (using a modified Jaffe reaction). All creatinine readings were above the manufacturer’s limit of detection of 0.042 mg/dL (to convert to micromoles per liter, multiply by 88.4). The intra-assay precision was 1.0%, and the interassay precision was 3.4%. To adjust for urinary dilution, all arsenic measures were normalized and reported as micrograms per gram of creatinine.

#### Subclinical CVD

Carotid-femoral pulse wave velocity assessed aortic stiffness following American Heart Association guidelines.^20^ The distances among the carotid pulse, sternal notch, and femoral pulse sites were measured as a straight line. Pulse waveforms from the carotid and femoral pulses were obtained sequentially with applanation tonometry and gated to the R-wave from simultaneous single-lead electrocardiography (Sphygmocor; Atcor Medical). Then cfPWV was calculated from the distances between measurement points and the measured time delay between proximal and distal pressure waveforms as follows: cfPWV = D/Δt (m⋅s^−1^).

Carotid intima media thickness was obtained from a longitudinal image of the common carotid artery obtained via vascular ultrasonography using a 7.5- to 10-MHz linear array probe (ProSound α7; Hitachi-Aloka). The IMT was measured on the far wall, 1 to 2 cm below the carotid bulb, as the distance from the lumen-intima interface to the media-adventitia interface across a 5-mm region of interest using semiautomated digital calipers.

Echocardiographic measurements were made using 2-dimensionally–directed M-mode echocardiograms using a cardiac ultrasound unit (Sonos 5500; Phillips). Measurements of diastolic and systolic left ventricular dimensions and septal and posterior wall thickness were made offline using a workstation (Digisonics Inc). Measurements were made according to the recommendations of the American Society of Echocardiography^21^ and demonstrated good 2-month test-retest reliability.^13^ These measurements were adjusted for height using the following formula that was validated in a pediatric sample: LVM index = LVM/[(height^2.16^) + 0.09].^22^

#### Patterns of Left Ventricular Geometry

Although LVM and RWT cut points used to define patterns of left ventricular remodeling (eg, concentric remodeling) are outlined in the American Society of Echocardiography’s recommendations for chamber quantification^23^ and used extensively in the adult literature,^24^ there are no recommendations for cut points in adolescents and children.^25^ Given that children in our sample were predominantly normotensive (Table), we used an 85th percentile cut point for determining relative elevations in LVM and RWT rather than a more conservative threshold of a 90th or 95th percentile.^16^ On the basis of these thresholds, we identified 4 groups: normal (normal LVM and RWT; n = 182), concentric remodeling (normal LVM and >85th percentile RWT; n = 23), concentric hypertrophy (>85th percentile LVM and >85th percentile RWT; n = 15), and eccentric hypertrophy (>85th percentile normal and normal; n = 24). Blood pressure was measured during the echocardiogram visit using an automated monitor (Dynamap).

**Table.** Characteristics of the Participants in the Study Sample

Measure	Full Sample (N = 245)	Girls (n = 133)	Boys (n = 112)
Child age, mean (SD), y	10.52 (0.93)	10.58 (0.95)	10.44 (0.91)
Race, No. (%)			
Black	145 (59.2)	78 (58.6)	67 (59.8)
White	100 (40.8)	55 (41.4)	45 (40.2)
BMI *z* score, mean (SD)	0.68 (1.28)	0.65 (1.18)	0.73 (1.24)
Systolic blood pressure, mean (SD), mm Hg	110.27 (8.76)	109.45 (8.27)	111.18 (9.15)
Diastolic blood pressure, mean (SD), mm Hg	62.80 (6.98)	63.10 (6.85)	62.34 (7.09)
Total urinary arsenic, mean (SD), μg/g creatinine	7.76 (0.53)^a^	7.96 (0.70)	7.71 (0.73)
Subclinical CVD indicators, mean (SD)			
Pulse wave velocity, m/s	4.58 (0.84)	4.62 (0.91)	4.52 (0.73)
Carotid IMT, mm	0.40 (0.06)	0.40 (0.06)	0.39 (0.07)
Left ventricular mass, g/m^2^	0.0019 (.0004)	0.0019 (0.0004)	0.0018 (0.0004)
Relative wall thickness ratio	0.30 (0.05)	0.30 (0.05)	0.31 (0.05)

Abbreviations: BMI, body mass index (calculated as weight in kilograms divided by height in meters squared); CVD, cardiovascular disease; IMT, intima media thickness.

^a^ Geometric mean.

### Exposure Assessments

#### Diet

Two days of children’s dietary data were collected from parents and children, with the help of a trained nutrition research assistant, using the Automated Self-Administered 24-Hour Dietary Assessment Tool, versions 2011 and 2016, developed by the National Cancer Institute.^26^ Data were averaged during the 2 days of intake for each participant. Food categories were then derived using the Healthy Eating Index.^27^

#### Other Potential Exposure Routes

Drinking water in Syracuse, New York, is all sourced from Skaneateles Lake, a lake with ongoing testing that demonstrates no significant elevation of arsenic.^28^ Given these low levels as well as the consistency in source across our cohort, drinking water was not considered a potential source for total arsenic in our cohort. We also considered parental reports of secondhand smoke exposure, assessed with a survey (0 indicating none; 1, <1 hour a day; 2, 1-5 hours a day; and 3, >5 hours a day). Finally, we used each participants’ home address to assess geographic clustering.

#### Covariates

The following covariates were included in all models: sex, age, race, SES, blood lead level (BLL), and BMI *z* score. Participant sex and race (self-identification as either Black or White) were obtained by self-report. Date of birth (ie, age) was reported by participants and confirmed by the parent or guardian. Height and weight were measured using a clinical scale (Detecto). For BMI, we used Centers for Disease Control and Prevention data and their provided SAS macro for age- and sex-adjusted BMI *z* scores.^29^ Parental reports of annual household income, occupation, and education level were *z* scored and averaged^30^ to form an SES index. On the basis of prior research demonstrating an association between BLLs and CVD,^31^ we included BLLs as an additional covariate in all models. Analytic methods for measurement of BLL in this cohort can be found in prior publications.^12,14^

### Statistical Analysis

Statistical analysis was performed from January 1, 2022, to February 28, 2023. Primary analyses were conducted using SAS software, version 9.4 (SAS Institute Inc). Although siblings were enrolled (N = 245 participants drawn from 199 families), effects of family clustering were minimal for cIMT (intraclass correlation = 0.29); therefore, more complex multilevel modeling was not necessary, yet primary findings were nevertheless confirmed in mixed-model analyses (eAppendix 2 in Supplement 1). For analysis of geographic patterns, we used a local Moran *I* test (LISA) to identify whether there were any spatial clusters among the participants’ total arsenic levels. LISA compares each observation with other nearby observations to identify whether there are any high values near other high values and whether low values are near other low values. We used analysis of covariance (ANCOVA) to group observations by school and household and tested whether those factors were associated with variation in clustering of high-high and low-low levels. A 2-sided *P* < .05 was considered statistically significant. All spatial analyses were completed in R statistical software, version 4.1.1 (R Foundation for Statistical Computing).

## Results

### Sample Characteristics

This sample included 245 children aged 9 to 11 years (mean [SD] age, 10.52 [0.93] years; 133 [54.3%] female and 112 [45.7%] male; 145 [59.2%] Black and 100 [40.8%] White) (Table). The sample had low-middle SES (eg, median family income was $25 000-$35 000 based on parental reports) as expected based on targeted zip codes. In a base model with only covariates entered, none of these sample characteristics were significantly associated with total arsenic levels. Total arsenic levels in our cohort were slightly higher (geometric mean, 7.76 μg/g in EECHO, creatinine adjusted) than the most relevant National Health and Nutrition Examination Survey (NHANES) comparison based on age and years of testing (geomatric mean, 7.08 μg/g in children aged 6 to 11 years living in the US in 2003 to 2004). The eTable in Supplement 1 reports age- and sex-adjusted partial correlations among all study variables.

### Total Arsenic and Subclinical CVD

#### cIMT and cfPWV

**Figure 1.**
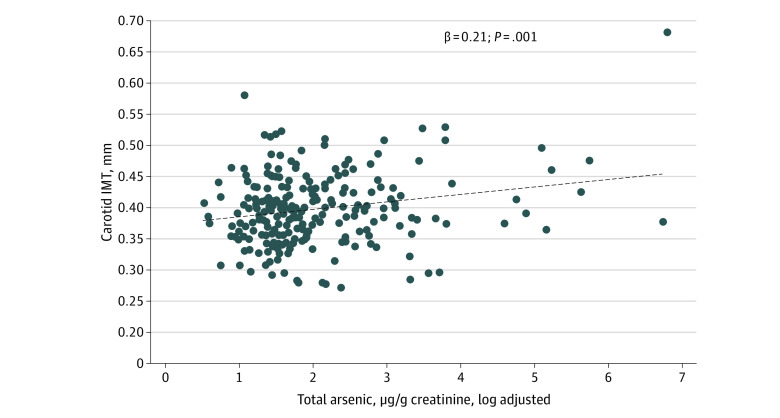
Carotid Intima Media Thickness (IMT) as a Function of Total Arsenic Adjusted for the Child’s Sex, Race, Age, Socioeconomic Status, Blood Lead Level, and Body Mass Index *z* Score

In regression models (n = 227 for cIMT and n = 214 for PWV), we considered whether total arsenic was associated with each subclinical CVD outcome. A simple regression model revealed that total arsenic (log-transformed) was associated with cIMT (β = 0.16 [95% CI, 0.03-0.28]; *t*_1,225_ = 2.40; *P* = .02). This association remained significant after controlling for race, sex, age, BLL, SES, and BMI (β = 0.17 [95% CI, 0.04-0.29]; *t*_1,219_ = 2.64; *P* = .009). Finally, in the full model with arsenic corrected for creatinine in urine, the association was still significant (β = 0.21 [95% CI, 0.08-0.33]; *t*_1,219_ = 3.27; *P* = .001) (Figure 1). The stepwise addition of quadratic (arsenic × arsenic) and cubic (arsenic × arsenic × arsenic) terms did not add significantly to the association with cIMT (quadratic: *t*_1,218_ = 1.84; *P* = .07; cubic: *t*_1,217_ = 1.39; *P* = .17). In addition, models with interaction terms revealed no significant difference in this total arsenic–cIMT association as a function of sex (*t*_1,218_ = 0.57; *P* = .57) or race (*t*_1,218_ = 1.62; *P* = .11). Total arsenic was not significantly associated with cfPWV (β = 0.07 [95% CI, −0.06 to 0.29]; *t*_1,212_ = 1.05; *P* = .29) in the full model.

#### Patterns of Left Ventricular Geometry

**Figure 2.**
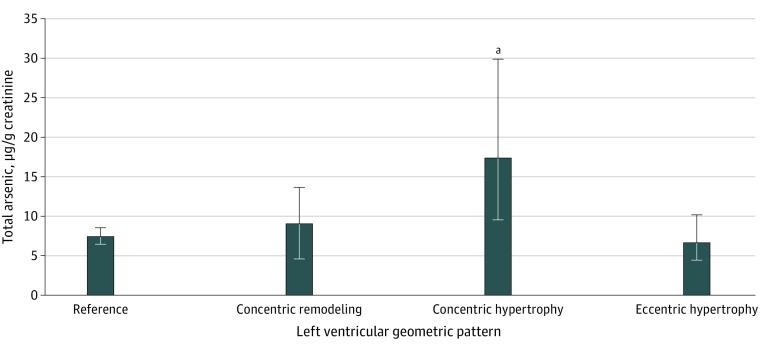
Geometric Mean Total Arsenic Levels as a Function of the Left Ventricular Geometric Pattern Adjusted for the Child’s Sex, Race, Age, Socioeconomic Status, Blood Lead Level, and Body Mass Index *z* Score (N = 244) Significance is reported for group differences using the Tukey test to correct for potential type I error with multiple comparisons. Although toxicant exposure levels were log transformed in analyses, for purposes of illustration we use original (creatinine-adjusted) units. Error bars indicate 95% CIs.

In an ANCOVA considering echocardiographic data (N = 244), total arsenic levels were associated with significantly different patterns of left ventricular geometry (*F*_3,234_ = 3.23; *P* = .02) (Figure 2). A Tukey test for multiple planned comparisons revealed significantly greater total arsenic levels for those in the concentric hypertrophy group (geometric mean, 16.77 μg/g creatinine; 95% CI, 9.87-28.79 μg/g) compared with the reference group (geometric mean, 7.39 μg/g creatinine; 95% CI, 6.36-8.58 μg/g). Although our analyses were conducted with ln-transformation of total arsenic, the means and CIs reported above and in Figure 2 are reported after conversion to original units.

### Potential Source of Arsenic in Urine

#### Parental Smoking and Drinking Water

Parents reported no secondhand smoke exposure for 168 participants (68.6%). After entering covariates, the linear contrast across secondhand smoke exposure groups was not significant (*F*_1,232_ = 0.00; *P* = .99). Drinking water was not considered a source of variability in arsenic exposure because nearly all Syracuse residents draw water from 1 well-tested source, Skaneateles Lake.

#### Dietary Intake

After covariates were entered, total arsenic levels were regressed on dietary consumption categories. No significant associations were observed for total arsenic and Healthy Eating Index food groups. However, from our arsenic speciation study, the following food groups were associated with organic arsenic levels in urine: total vegetables (β = −0.46; *P* = .03), greens and beans (β = −0.60; *P* = .003), total fruit (β = 0.45; *P* = .04), and seafood and plant proteins (β = −0.66; *P* = .001). Together, these 4 food groups accounted for 51.2% (*R*^2^) of the variability in organic arsenic. Consistent with a seafood source for organic arsenic in this population, total grams consumed by this population within the seafood and plant protein group included the following: 48.1% seafood; 38.9% beans, peas, and legumes; and 13.0% nuts and seeds. For inorganic arsenic, only whole fruit (β = −0.46; *P* = .04) and total fruit (β = −0.38; *P* = .08) were associated with inorganic arsenic and accounted for only 9.8% (*R*^2^) of the variability in inorganic arsenic.

#### Geographic Clustering of Total Arsenic Levels

**Figure 3.**
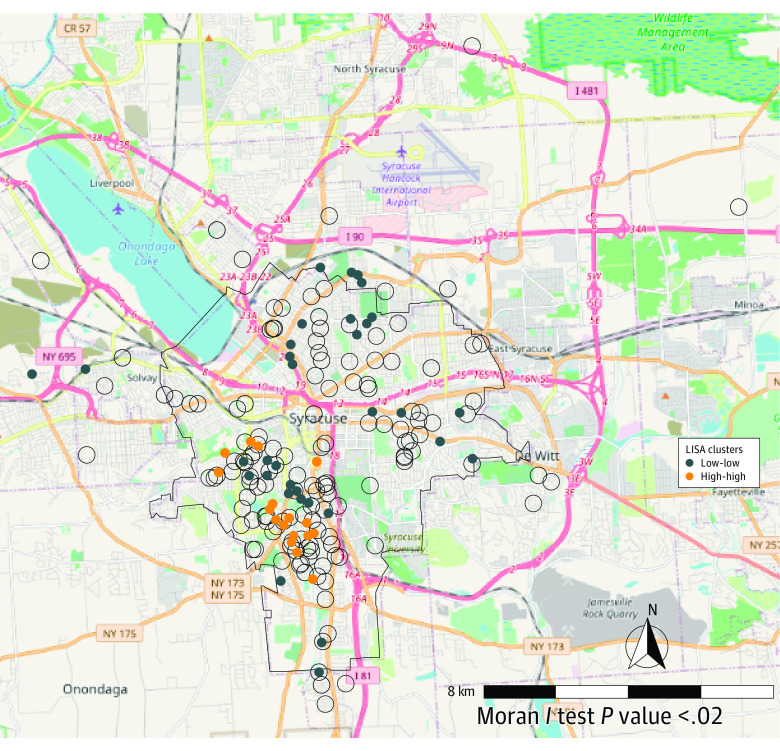
Geographic Clustering of Arsenic Levels Using Children’s Home Address LISA indicates local Moran *I* test.

The LISA method detected a cluster of high-high total arsenic levels among participants in the southwest side of Syracuse (Figure 3). The mean LISA statistic for high-high observations was 0.49, with a mean pseudo *P* = .09, suggesting a statistically significant geographic cluster of high total arsenic levels among participants in that area. There were also clusters of low-low values in the southwest side of Syracuse, but most low-low clusters were on the northern side of the city (Figure 3). The mean LISA statistic for low-low observations was 0.23, with a mean pseudo *P* = .10, suggesting statistically significant clusters of low total arsenic levels among participants in those areas. Participants not found to be in clusters of high-high or low-low values (n = 124) had a mean LISA statistic of 0.02 and mean pseudo *P* = .34 for nonsignificant clusters. In addition, the ANCOVA results did not find any association between the schools the children attended and whether the participant was in a cluster. In addition, the ANCOVA results found no association between whether participants were from the same household and whether they were in a cluster of high-high or low-low total arsenic levels.

## Discussion

Our hypothesis in this cross-sectional study was partially confirmed: we found a significant positive association between 1 indicator of subclinical CVD, cIMT, and total arsenic in children. This association did not differ significantly as a function of race, SES, or sex, which may indicate that total arsenic exposure is attributable to some other factor, such as geographic location. Furthermore, children exhibiting concentric hypertrophy (increased RWT and LVM) had significantly higher levels of total arsenic than the reference group (normal RWT and LVM). We believe our study represents the first to date to identify an association between actual body burdens of arsenic (in this case, total arsenic) and subclinical CVD in children.

We also consider potential sources of arsenic exposure in this cohort. Neither water nor secondhand smoke exposure was found to contribute to total arsenic in this cohort. Organic arsenic but not the inorganic component of total arsenic appears to be diet driven in our cohort. However, a significant cluster of elevated total arsenic was found in a specific area of Syracuse that is well known for industrial pollution,^32,33,34^ likely a function of being southeast of Onondaga Lake, an area aligned with prevailing winds and watershed runoff. This area is currently a superfund site as a result of the industrial and municipal sewage discharge for more than 100 years,^35^ suggesting this as the likely route of exposure to inorganic arsenic species in our cohort. Residents of this area are predominantly Black; as such, this location has also been the focus of continued concerns with environmental justice.^36^ Total arsenic levels in our cohort were elevated compared with national levels (NHANES),^37^ suggesting that this area in Syracuse might represent a hot spot for arsenic exposure.

### Limitations

There are a few limitations to the current study. First, the cross-sectional design keeps open the possibility that unmeasured variables covary with total arsenic and subclinical CVD, serving as uncontrolled confounding variables. For example, arsenic exposure in children may be greatest in disadvantaged communities,^38^ where children face additional adversity (eg, violence, maltreatment, homelessness, and parental death) that, in turn, affect CVD risk in adulthood.^39^ Our inclusion of SES as a control variable provides some protection from this alternative explanation; however, it does not capture the entire experience of these children. Second, we considered only subclinical CVD given the population (children). As such, although total arsenic levels appear associated with subclinical disease in childhood, neither subclinical CVD in childhood nor childhood arsenic exposure has yet been shown to be associated with actual clinical disease in adulthood. Third, given that variability in organic arsenic contributes to variability in total arsenic in the current population, particularly in children with high total arsenic levels, our findings may be underestimating the association of arsenic with disease end points in children.

## Conclusions

Our primary finding was that children’s total arsenic levels were significantly associated with subclinical CVD as indexed by cIMT and concentric cardiac hypertrophy. If further research suggests this association is causal, this finding would have important implications for future efforts at environmental remediation of this ubiquitous toxicant. Notably, total arsenic levels in this cohort do not appear to be associated with drinking water or secondhand smoke but rather are driven by diet for organic arsenic and, given the geographic clustering in an area with long-standing industrial pollution in Syracuse, likely air and soil contamination for inorganic arsenic exposure in this cohort.

## Notes

Supplement 1. **eAppendix 1.** Supplemental Methods **eAppendix 2.** Supplemental Results **eTable.** Partial Correlations (Controlling for Age and Sex) and Bivariate Sample Size (N) Among Study Variables

Supplement 2.
Data Sharing Statement
