# Supplementary material for: Exposure to Arsenic and Subclinical Cardiovascular Disease in 9- to 11-Year-Old Children, Syracuse, New York
Source: JAMA Netw Open. 2023 Jun 30;6(6):e2321379. doi: 10.1001/jamanetworkopen.2023.21379 (PMC10314305; doi:10.1001/jamanetworkopen.2023.21379)
Supplement: Supplement 1. — eAppendix 1. Supplemental Methods eAppendix 2. Supplemental Results eTable. Partial Correlations (Controlling for Age and Sex) and Bivariate Sample Size (N) Among Study Variables [file jamanetwopen-e2321379-s001.pdf]

Supplementary Online Content

Gump BB, Heffernan K, Brann LS, et al. Exposure to arsenic and subclinical cardiovascular disease in 9- to 11-year-old children, Syracuse, New York. *JAMA Netw Open*. 2023;6(6):e2321379. doi:10.1001/jamanetworkopen.2023.21379

eAppendix 1. Supplemental Methods
eAppendix 2. Supplemental Results
eTable. Partial Correlations (Controlling for Age and Sex) and Bivariate Sample Size (N) Among Study Variables

This supplementary material has been provided by the authors to give readers additional information about their work.

© 2023 Gump BB et al. *JAMA Network Open.*

eAppendix 1. Supplemental Methods

Participants

With respect to joint participation from the same family, 152 children participated alone, 84 children participated with 1 sibling, and 9 children participated with 2 siblings. As we describe below, the data analysis strategies reflected the nested nature of the data.

Statistical Analyses

Because some siblings participated from the same family and therefore had a shared family environment, ancillary analyses were conducted using PROC MIXED to test mixed models with fixed and random effects. In these models, covariates were treated as fixed factors and we used a single random factor to account for any family nesting effects among the siblings participating in the study. The covariance structure was set to the default, variance components (VC).

© 2023 Gump BB et al. *JAMA Network Open.*

eAppendix 2. Supplemental Results

Urine As and Subclinical CVD

cIMT and cfPWV. When tested using a mixed model approach to account for family nesting effects, the association between urine As and cIMT was nearly unchanged and remained significant (t (32) = 2.61, p = .014). As was not significantly associated with cfPWV in mixed models (t (28) = 1.08, p = 0.289).

Left Ventricular Geometric Patterns. As shown in Figure 2, urine As levels were again associated with significantly different patterns of LV geometry when analyzed with mixed models , t (27) = 2.58, p = .016. When tested using a mixed model approach to account for family nesting effects, the difference in As between the reference group and concentric hypertrophy group remained significant (t (36) = 2.41, p = 0.021).

© 2023 Gump BB et al. *JAMA Network Open.*

eTable. Partial Correlations (Controlling for Age and Sex) and Bivariate Sample Size (N) Among Study Variables

**Measures**	**1**	**2**	**3**	**4**	**5**	**6**	**7**	**8**	**9**	**10**	**11**
1. Race (0=Black; 1=White)	1.00	--	--	--	--		--	--	--	--	--
2. BMI (z score)	-0.12 (245)	1.00	--	--	--		--	--	--	--	--
3. Blood Lead Level (BLL)	-0.13 * (245)	-0.18 ** (245)	1.00	--	--		--	--	--	--	--
4. SES score	0.30 *** (245)	-0.11 (245)	=0.24 *** (245)	1.00	--		--	--	--	--	--
5. Creatinine	-0.16 * (245)	=0.19 ** (245)	0.04 (245)	-0.01 (245)	1.00		--	--	--	--	--
6. Second hand smoke	-0.12 (242)	0.16 * (242)	0.18 ** (242)	-0.32 *** (242)	0.04 (242)	1.00					
7. t-As (µg/g) in urine	-0.02 (245)	-0.19 ** (245)	0.08 (245)	-0.05 (245)	0.21 ** (245)	-0.00 (245)	1.00	--	--	--	--
8. cIMT	-0.25 *** (227)	0.18 ** (227)	-0.02 (227)	-0.02 (227)	-0.02 (227)	0.00 (224)	0.07 (227)	1.00	--	--	--
9. PWV	-0.09 (221)	0.21 ** (221)	0.04 (221)	-0.12 (221)	0.09 (221)	0.13 (218)	0.14 * (221)	0.03 (217)	1.00	--	--
10. LV mass	-0.17 ** (244)	0.56 *** (244)	-0.00 (244)	-0.09 (244)	=0.12 * (245)	0.00 (241)	-0.13 * (244)	0.18 ** (227)	0.07 (221)	1.00	--
11. RWT	-0.13 * (244)	0.19 ** (244)	0.15 * (244)	=0.16 * (244)	-0.10 (244)	0.03 (241)	0.03 (227)	0.15 * (227)	-0.07 (221)	0.41 *** (244)	1.00

© 2023 Gump BB et al. *JAMA Network Open.*
