# Supplementary material for: Exposure to Arsenic and Subclinical Cardiovascular Disease in 9- to 11-Year-Old Children, Syracuse, New York
Source: JAMA Netw Open. 2023 Jun 30;6(6):e2321379. doi: 10.1001/jamanetworkopen.2023.21379 (PMC10314305; doi:10.1001/jamanetworkopen.2023.21379)
Supplement: Supplement 2. — Data Sharing Statement [file jamanetwopen-e2321379-s002.pdf]

Data Sharing Statement

Gump. Exposure to Arsenic and Subclinical Cardiovascular Disease in 9- to 11-Year-Old Children, Syracuse, New York. *JAMA Netw Open*. Published June 30, 2023. doi:10.1001/jamanetworkopen.2023.21379

Data
Data available: No

Additional Information
Explanation for why data not available: Study analyses are ongoing.
